# Achieving a Good Response in Myxofibrosarcoma With Uncontrollable Bleeding Using an Enzyme-Targeting Radiosensitization Treatment: A Case Report

**DOI:** 10.7759/cureus.77800

**Published:** 2025-01-21

**Authors:** Kana Kobayashi, Yasuhiro Ogawa, Ryosuke Bessho, Hironori Tanaka, Ryohei Sasaki

**Affiliations:** 1 Division of Radiation Oncology, Kobe University Hospital, Kobe, JPN; 2 Radiology, Kochi University, Kochi, JPN; 3 Radiation Oncology, Hyogo Cancer Center, Akashi, JPN

**Keywords:** bleeding, enzyme-targeting radiosensitization, external beam radiotherapy, hydrogen peroxide, kortuc, myxofibrosarcoma, tumor hypoxia

## Abstract

Myxofibrosarcoma is one of the most prevalent histological types of primary malignant soft tissue tumors of the extremity. Radiation therapy is frequently employed as a post-operative treatment. In this case report, we present a 78-year-old male with a large tumor on his forearm. He refused surgical treatment. Over a period of seven years, recurrence and bleeding were repeatedly observed. Kochi oxydol radiation therapy for unresectable carcinomas (KORTUC) is a treatment modality that uses a hydrogen peroxide solution that adheres to the tumor surface (KORUTC I), while the solution is also injected into the tumor (KORTUC II). We treated the tumor with KORTUC I and II. A good response was observed. This case report highlights the effectiveness of KORTUC I and II. Reports on KORTUC for sarcomas are limited, underscoring the necessity for future examination of cases not indicated for surgical intervention.

## Introduction

One of the characteristics of solid tumors that leads to radioresistance is the presence of a hypoxic environment and antioxidant enzymes. The efficacy of general X-ray therapy for relatively large solid tumors is known to be reduced by approximately one-third due to the dominant presence of hypoxic cells [1].

When tumors enlarge, the environment inside the tumor becomes hypoxic, and antioxidative enzymes such as peroxidase and catalase increase, making radiotherapy much less effective. This has been a fundamental problem in radiotherapy and/or chemotherapy and has remained unresolved for a long time, especially for large tumors, including soft tissue sarcomas.

Kochi oxydol radiation therapy for unresectable carcinomas (KORTUC) is a treatment modality that uses a hydrogen peroxide solution that adheres to the tumor surface (KORUTC I) [2], while the solution is also injected into the tumor (KORTUC II) [3].

To improve the tumor micro-environment from becoming resistant to radiotherapy, we found a powerful way to eliminate tumor hypoxia and inactivate antioxidative enzymes by injecting KORTUC (a mixture of hydrogen peroxide and sodium hyaluronate) straight into the tumor twice a week during the period of radiation therapy. 

In recent years, there has been growing interest in enzyme-targeted and sensitized radiotherapy [4]. The standard administration of KORTUC II is twice weekly [2,3]. It was reported that the concentrations of oxygen and hydrogen peroxide undergo a significant decline after 24 hours [5]. In radiobiology, irradiation has direct and indirect effects. The effect of X-rays and electron beams is mainly indirect effects. Without oxygen molecules, radiation would have no indirect effects. Therefore, the oxygen concentration within the tumor is considered important because it affects the antitumor effect. However, the extent of this decline in concentration is believed to exert minimal influence on the therapeutic outcome until reaching 72 hours [6]. Although oxygen concentration within the tumor decreases, a sufficient amount remains at 72 hours, indicating that the therapeutic effect is maintained. Consequently, the protocol is implemented on a biweekly basis.

Although KORTUC has only been used in large clinical trials for breast cancer [7], its efficacy has been extensively documented since 2008 [2]. The theoretical background is as follows: the antioxidant enzyme peroxidase, which is naturally present in high levels in many cancerous tissues, is inactivated by the addition of hydrogen peroxide, which then decomposes into water and oxygen. KORTUC facilitates the decomposition of antioxidant enzymes, resulting in the production of oxygen. Injection of the agent into the cancerous tissue results in the inactivation of the peroxidase enzyme within the cell, leading to an increase in hydrogen peroxide. This is caused by the accumulation of hydroxyl radicals generated by radiotherapy within the lysosome, which ultimately induces lysosome-derived apoptosis [4,8]. The efficacy of KORTUC has been observed in a range of other tumor types, including malignant melanoma [2], pancreatic cancer [9], and pelvic sidewall recurrence of cervical cancer [10]. In this study, we present a case in which KORTUC was used to preserve the affected limb in a patient with refractory myxofibrosarcoma who had previously undergone surgical treatment but had not responded.

## Case presentation

A 78-year-old male presented with a mass on the extensor side of his right elbow. Seven years prior to our treatment, he had been diagnosed with dedifferentiated liposarcoma, and he requested follow-up appointments. Six years earlier, the tumor showed growth, and subsequent cytological analysis led to a diagnosis of myxoid liposarcoma. However, the patient requested continued follow-up monitoring. Over the next three years, the patient exhibited progressive disease, including increased mass size and pain intensity. A needle biopsy revealed a diagnosis of myxofibrosarcoma based on the following findings by a pathologist: the specimen contained spindle-shaped, star-shaped atypical cells growing against the background of myxomatous stroma. There was no high degree of atypia or polymorphism, nor was there dense tumor growth. The fission images were rare, but the Ki-67 labeling index (LI) is high at about 40%. Immunostaining showed negative results for AE1/3, EMA, αSMA, desmin, s-100, and MUC-4, and positive for CD-34 in some cases. 

Despite this diagnosis, the patient expressed a strong desire to preserve the affected limb and opted for conservative treatment. Pazopanib was initiated at that time; however, all systemic therapies resulted in progressive disease, and neither doxorubicin nor eribulin nor trabectedin were effective. Approximately two months prior to the initial visit to our hospital, anemia resulting from tumor-related bleeding necessitated blood transfusions and palliative partial tumor resection of the right elbow on two occasions.

**Figure 1:**
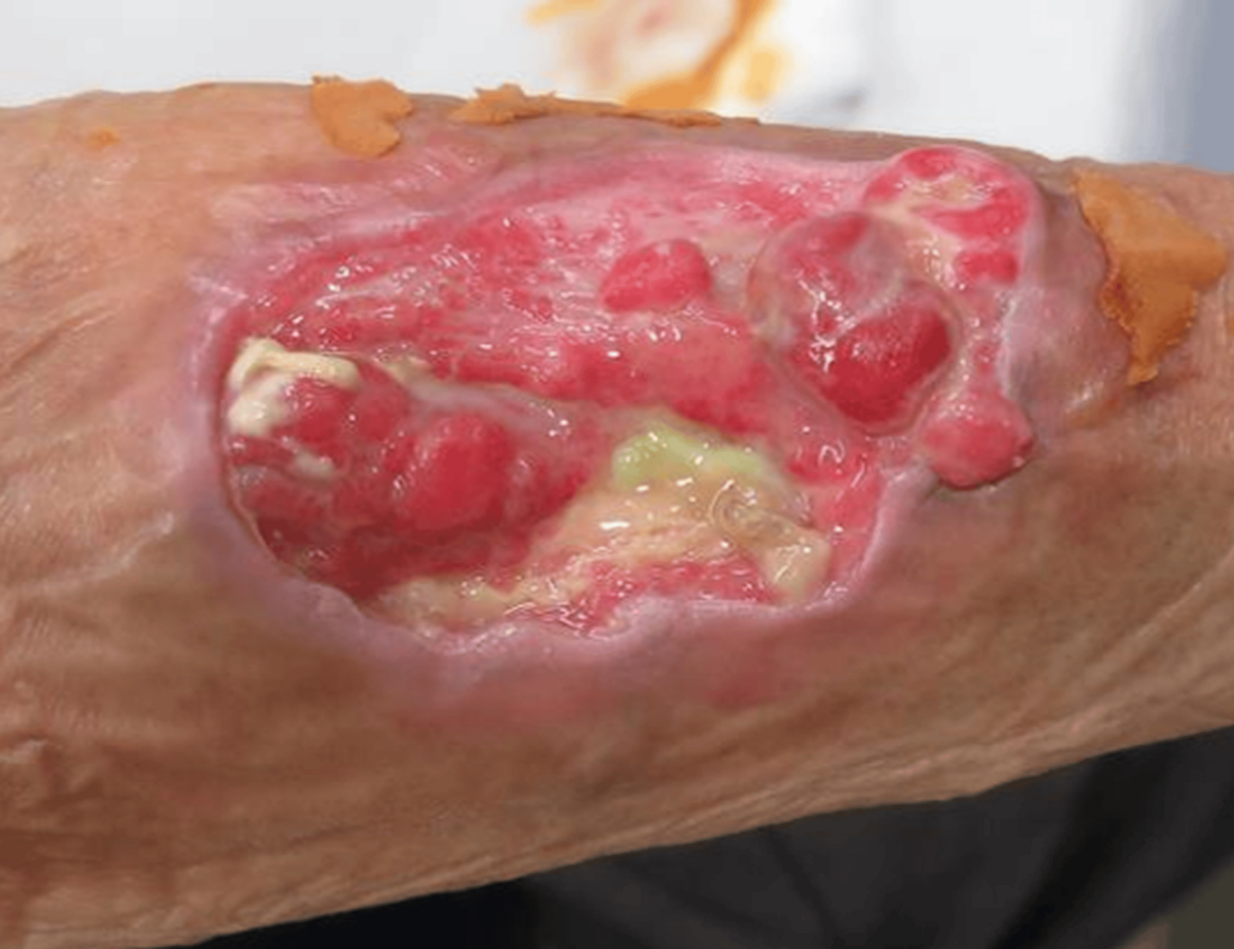
Myxofibrosarcoma patient at his first visit to our hospital

At the time of the preliminary physical examination, the tumor was measured at 6.0 × 7.6 × 0.5 cm³ and was located at the right forearm. The tumor was accompanied by skin defects and ulcerations, as shown in Figure 1. On the forearm side, bleeding from the elevated lesion was observed, and the patient primarily complained of difficulty using the right hand. Concurrently, blood tests were conducted, revealing mild anemia. A PET-CT scan was performed, but no metastasis was observed. Chemotherapy had already been administered, but no effective treatment had been identified. Surgery was recommended once again, but the patient refused because the surgical indication was only amputation. Carbon ion radiotherapy was subsequently considered, but it was deemed contraindicated due to the risk of adverse events outweighing the therapeutic benefit, as the probability of severe dermatitis was high, and even if the irradiation were to be effective, the skin would be difficult to regenerate, and ulcers would persist. Palliative irradiation was also considered; however, the absence of distant metastases, the patient’s generally good condition, and his preference for high-intensity local treatment led to the decision to utilize radiotherapy in combination with KORTUC.

According to Japan’s insurance coverage, the utilization of oxidol (3% hydrogen peroxide) is restricted to the disinfection of superficial wound lesions on the skin’s surface. Its insurance coverage is not intended for close contact with the tumor, such as KORTUC I, or for the injection of the agent into deep body tissues, such as KORTUC II. Consequently, treatment was initiated after receiving approval from the Ethical Review Committee of Kobe University, Kobe, Japan.

**Figure 2:**
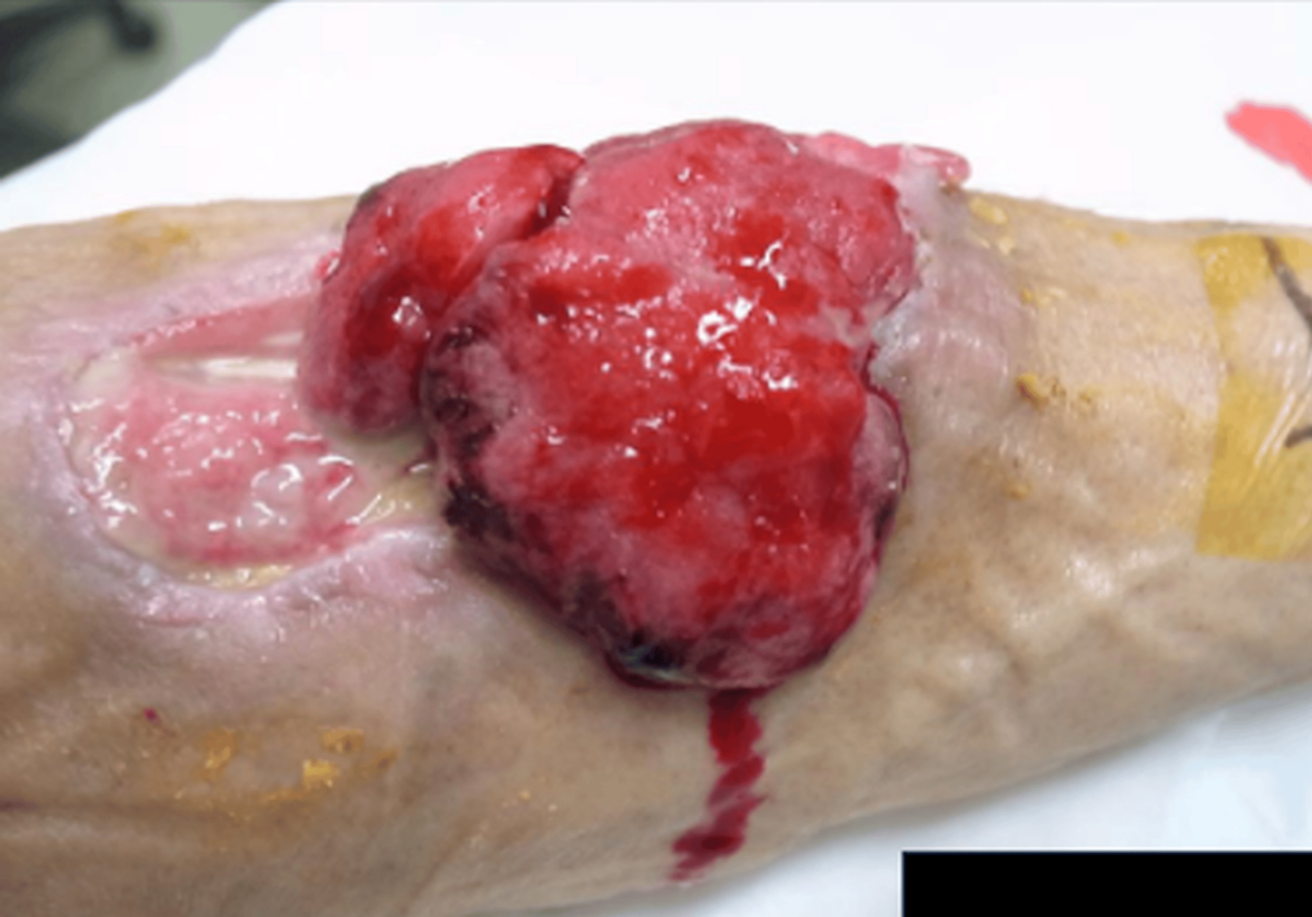
Pre-treatment status of the tumor two months after the first visit

**Figure 3:**
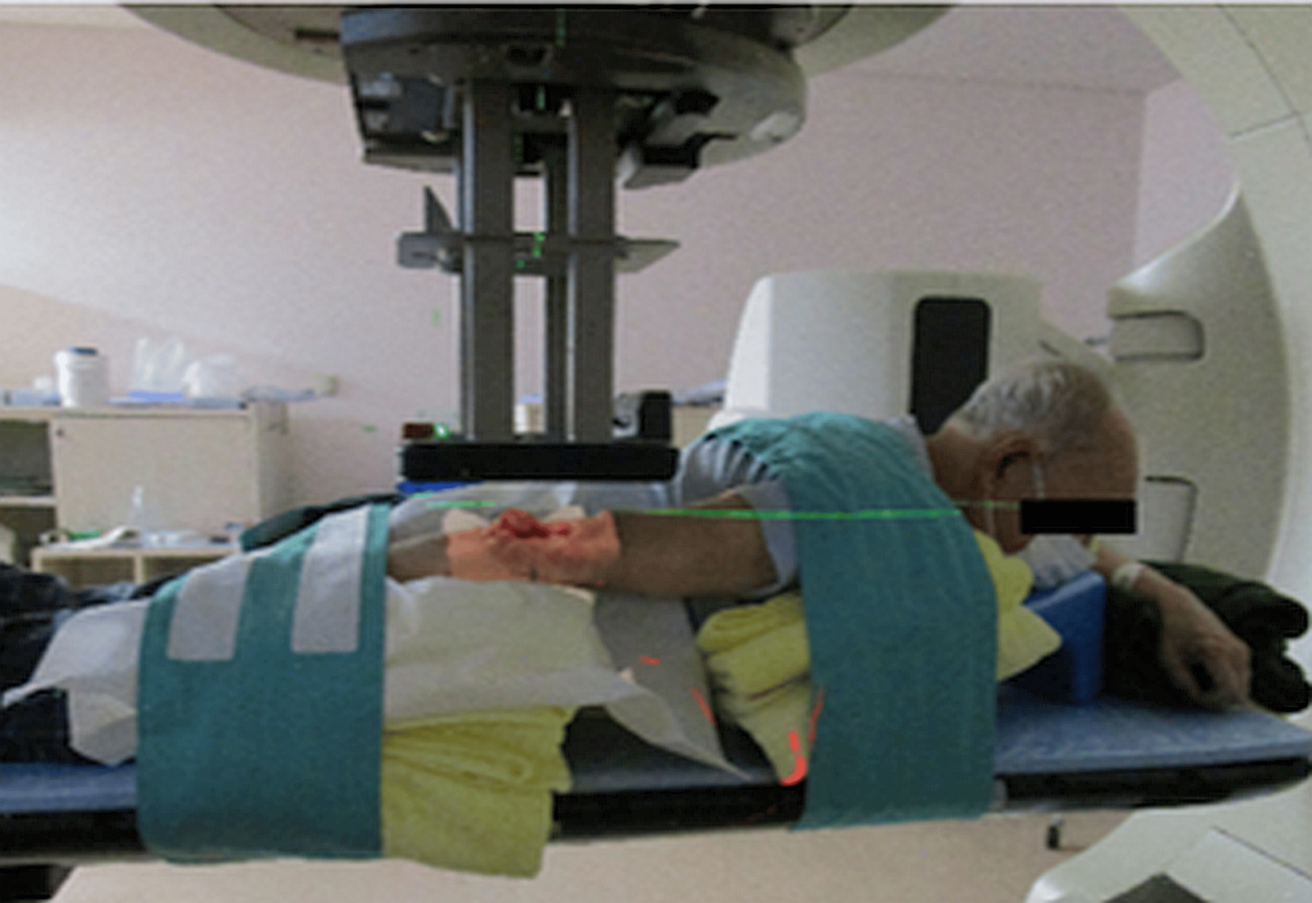
Setup of the electron beam therapy

**Figure 4:**
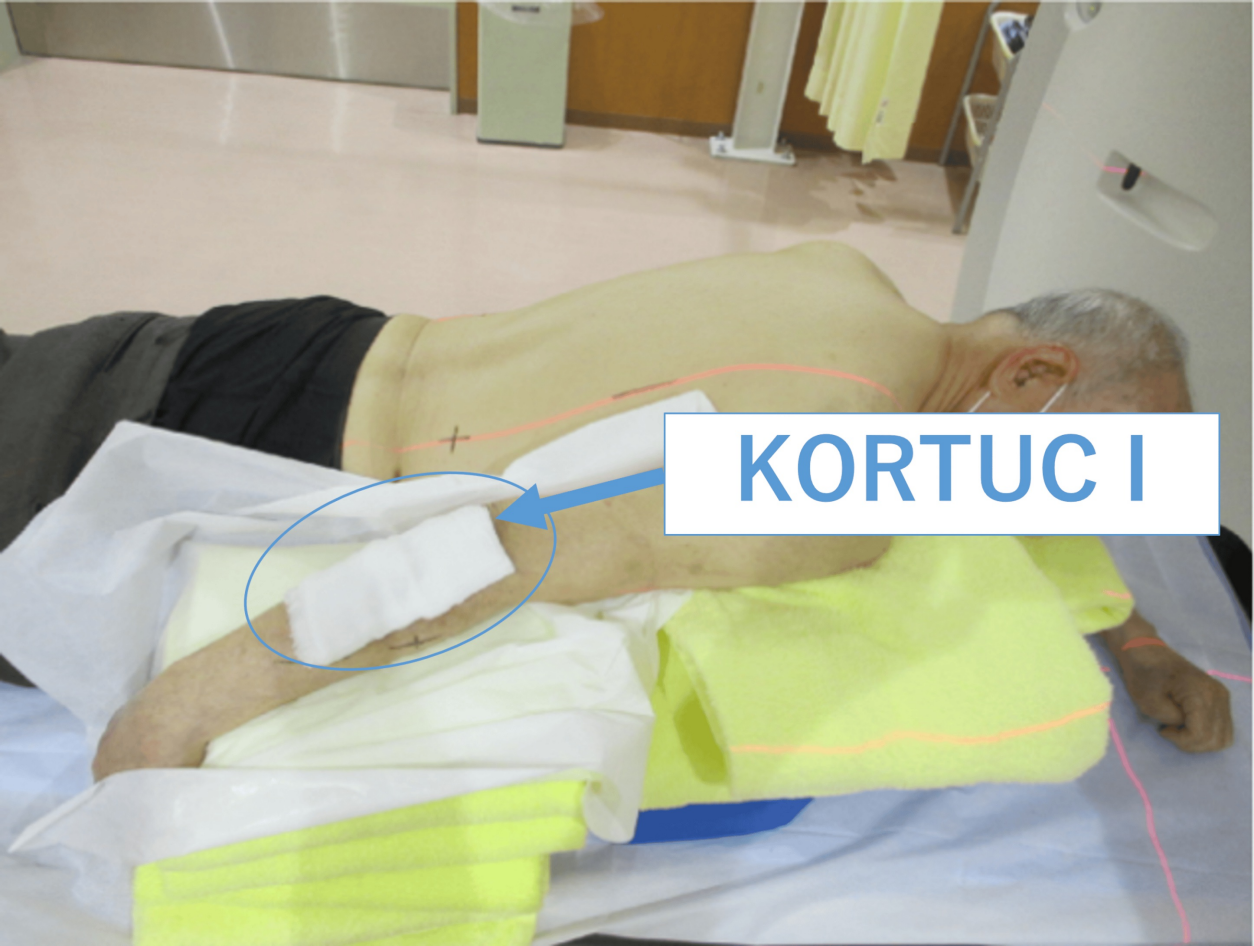
KORTUC I Kochi oxydol radiation therapy for unresectable carcinomas (KORTUC) is originally a treatment that uses a hydrogen peroxide solution-soaked gauze bolus that covers the superficially exposed tumor surface (KORTUC I). Since it is sufficiently immersed in hydrogen peroxide solution, it also provides a bolus dose enhancement and exhibits an antitumor effect as the tumor reacts with hydrogen peroxide solution during KORUTC.

**Figure 5:**
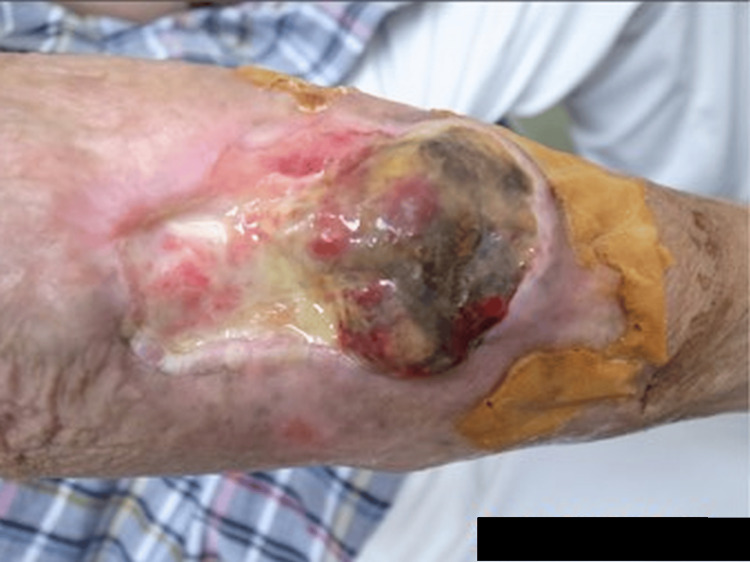
Post-treatment observation One month after the treatment, the tumor decreased. The ulcer lesion from which the tumor was exited was smaller with regeneration of skin from the edge.

**Figure 6:**
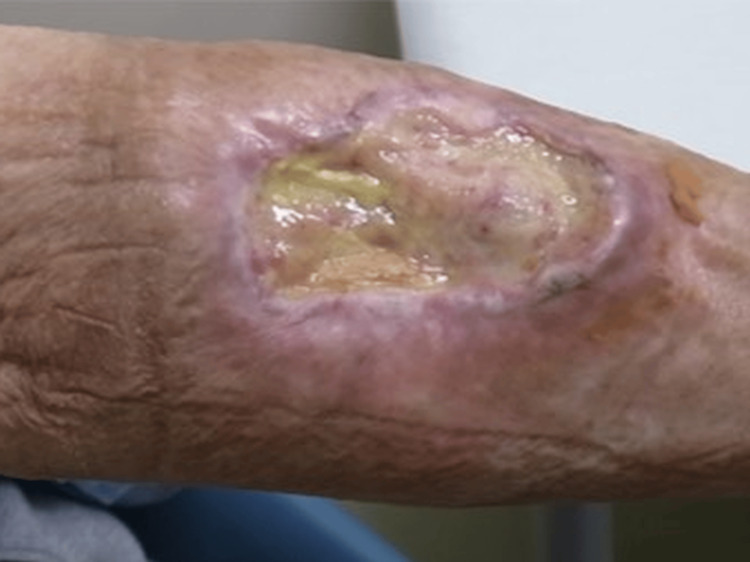
Four months post-treatment

The ethical review process required two months. During this period, the tumor grew, exhibiting skin defects and ulcerations measuring 6.0 × 7.6 × 2.5 cm³ from the distal right arm to the proximal forearm. Bleeding was observed from the elevated lesion on the forearm side, as shown in Figure 2. We anticipated that the ethics committee review could take at least two months, so there would be an increase in the risk of bleeding during that time. Since the patient lived far away from our hospital, we asked the local dermatologist and the medical oncology to provide care. For the local dermatologist, we asked to prescribe an ointment for bleeding, and for the medical oncologist, we asked to transfuse blood in the event of anemia. Given the tumor’s relatively thin profile, a method of electron beam irradiation was considered (Figure 3), using hydrogen peroxide gauze called KORTUC I, as indicated in Figure 4. However, due to the observed growth of the tumor, a different approach, namely KORTUC II, was also adopted. Electron beam irradiation was performed at 9 MeV with a prescribed dose of 60 Gy in 20 fractions in four weeks. KORTUC I was administered in each fraction, while KORTUC II was injected into the tumor on a biweekly basis. Each injection involved the administration of two vials, with each containing 0.5 mL of oxidol (3% hydrogen peroxide) and 2.5 mL of hyaluronic acid. This process was repeated seven times, with the injection being administered immediately prior to irradiation, as demonstrated in Video 1. The injection was associated with mild pain, but no anesthesia was required. The only treatment-emergent adverse event was grade 1 dermatitis, and no other significant adverse events were observed. By the end of the treatment course, the bleeding had stopped, as shown in Figure 5. Four months after the treatment, the tumor disappeared, as shown in Figure 6, and the affected right hand demonstrated a complete recovery in functionality. Unfortunately, he contracted COVID-19 and died of pneumonia shortly before the six-month post-treatment follow-up, and his subsequent course could not be monitored.

**Video 1:** KORTUC Ⅱ KORTUC, Kochi oxydol radiation therapy for unresectable carcinomas

## Discussion

Myxofibrosarcoma represents one of the most prevalent histological types of primary limb sarcoma [11]. The propensity for infiltration is pronounced, and the local recurrence rate following surgical intervention is as high as 32-60% [12]. As with other sarcomas, this tumor is resistant to radiation. Radiation therapy is frequently employed as a post-operative treatment. Histologically, the tumor can be classified into three categories: low, intermediate, and high grade, with the grade worsening with recurrence. The histological grade of myxofibrosarcoma is known to deteriorate with repeated recurrences; therefore, it is probable that the patient was of a high grade at the time of referral in view of his history. There are few reports of KORTUC for bone and soft tissue tumors; however, successful responses to KORTUC were reported in cases of superficial lesions [2]. There is a paucity of documented cases of rapid growth that resemble the observations made in this patient. Our findings suggest that, even in cases of high-grade myxofibrosarcoma with limited treatment options, the combination of KORTUC I and II may be effective in achieving hemostasis and tumor control.

Carbon ion radiotherapy has also been shown to be an effective treatment for bone and soft tissue tumors. However, tumors located on the surface of the body are contraindicated for treatment due to the potential for intractable ulceration. Given the rapid growth of the tumor prior to treatment, the use of a single beam of electron or X-ray to respond to changes in tumor size can facilitate the rapid creation of an appropriate irradiation plan and the continuation of treatment. However, in cases such as IMRT, the treatment planning process is time-consuming, and during that period, there is a high probability that the tumor’s shape will undergo changes, potentially leading to discrepancies in the calculations. This necessitates the process of replanning. In this particular instance, during the irradiation process, the tumor’s size gradually decreased from the margin. There was no sudden increase after the commencement of treatment, and the maximum thickness of the tumor remained constant, thereby precluding the need for replanning. The only adverse event observed was grade 1 radiation dermatitis, and bleeding also ceased. There is no established appropriate treatment procedure for such cases [13].

In the absence of regulatory approval in Japan, hydrogen peroxide is a drug permitted only for the disinfection of body surfaces. It took two months to receive permission to perform the treatment. In addition to ethical review, there are a number of hurdles to implementation. For example, since the treatment is not well known, it was necessary to fully explain the treatment to inexperienced medical practitioners and gain appropriate cooperation. Each facility had different detailed rules, and it was necessary to set various rules. It is hoped that in the future, it will be made into a drug, and these hurdles will disappear, and treatment will become more widespread. The tumor grew during the waiting period, but after treatment, in the end, the tumor disappeared, the bleeding stopped, and the patient's wishes were met. The patient’s pain was mild due to the absence of nerves within the tumor; therefore, xylocaine was not administered.

Unfortunately, the patient was infected with COVID-19 and died before a sufficient follow-up period, so treatment efficacy and late adverse events could not be evaluated. However, we believe that these results suggest that KORTUC treatment for inoperable myxofibrosarcoma may be effective.

Reports on KORTUC for sarcomas are limited, underscoring the necessity for future examination of cases not indicated for surgical intervention.

## Conclusions

In this case study, KORTUC was shown to be an effective treatment for myxofibrosarcoma, a challenging disease to treat. Unfortunately, the patient was infected with COVID-19 and died before a sufficient follow-up period, so treatment efficacy and late adverse events could not be evaluated. This was a limitation. However, we believe these results suggest that KORTUC treatment for inoperable myxofibrosarcoma may be effective. Sarcoma treatment with X-rays alone is challenging, and the potential for KORTUC to serve as a curative treatment is a promising avenue for further investigation.
